# Supplementary material for: Benefits of Polydopamine as Particle/Matrix Interface in Polylactide/PD-BaSO4 Scaffolds
Source: Int J Mol Sci. 2020 Jul 31;21(15):5480. doi: 10.3390/ijms21155480 (PMC7432262; doi:10.3390/ijms21155480)
Supplement: Supplementary file 1 [file ijms-21-05480-s001.pdf]

International Journal of
Molecular Sciences

MDPI

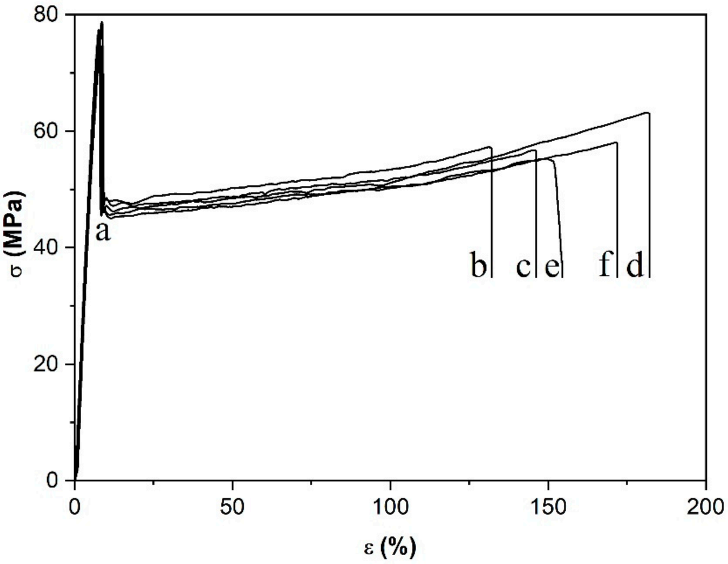

Figure S1. Tensile stress–strain curves of bulk materials (a) unfilled PLA, and PLA composites with (b) 0.5, (c) 1.0, (d) 2.0, (e) 5.0 and (f) 10.0 wt.% PD-$BaSO_4$.

The increase in elongation is given by the amount of particle in the composite, obtaining a maximum elongation for the 2 wt.% composition. Despite increasing the ductility of the material, the strength is not affected.

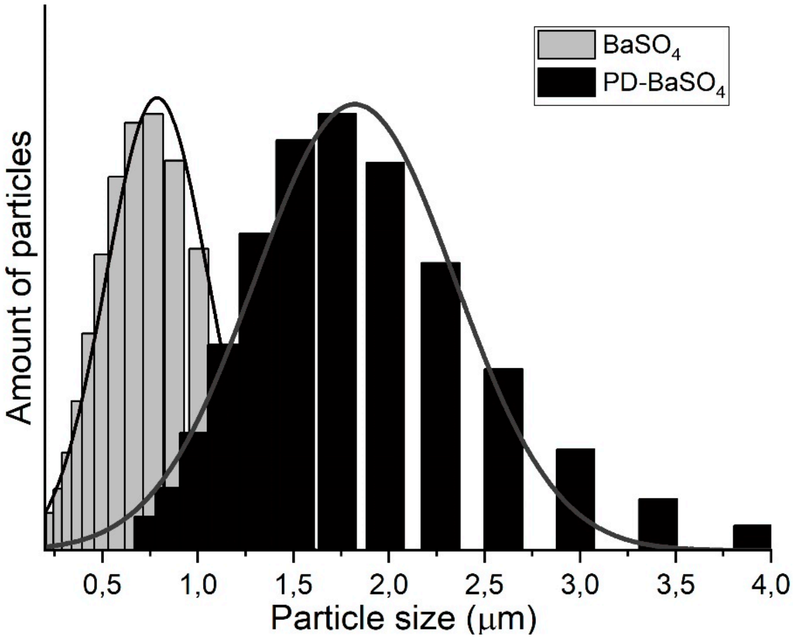

Figure S2. Particle size of $BaSO_4$ (Grey) and PD-$BaSO_4$ (black)

In the case of $BaSO_4$, a particle size between 0-2 μm is determined, with a maximum of 0.75-1 μm, that expresses the average size. As expected, the polydopamine (PD) coated particles present a distribution of particle sizes displaced to higher values and showing a maximum between 1.5-2 μm.

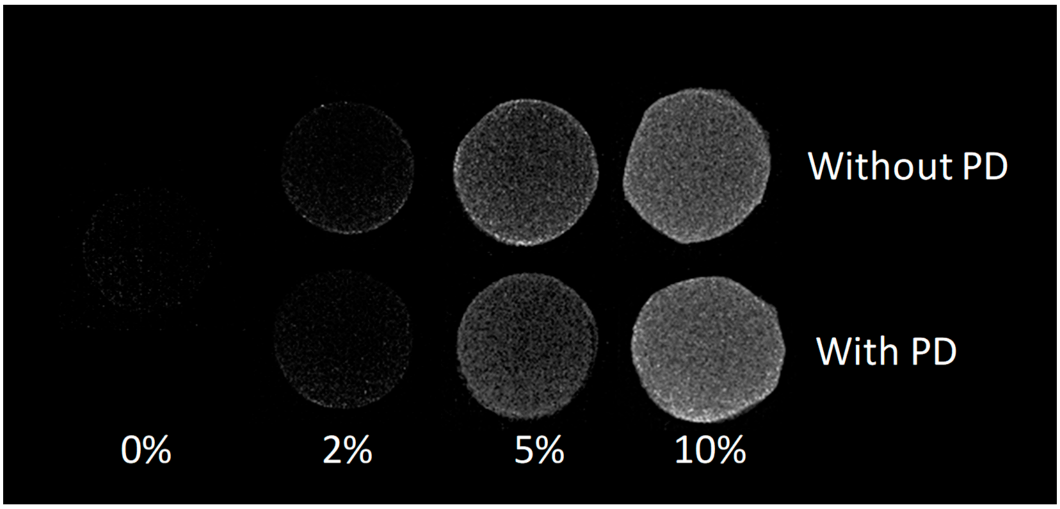

Figure S3. X-radiographies for neat PLA, PLA/$BaSO_4$ and PLA/PD-$BaSO_4$ composites with 2, 5 and 10 wt. % of $BaSO_4$ and PD-$BaSO_4$

It is observed how barium sulfate provides radiopacity to neat PLA, and how the polydopamine coating is not affecting the radiopacity level. In both cases, it is seen that above 5 wt. % $BaSO_4$ and PD-$BaSO_4$ polylactide composites express adequate radiopacity, thus for a clinical application clinical application 10 wt. % BaSO4 PLA composites will be preferred.

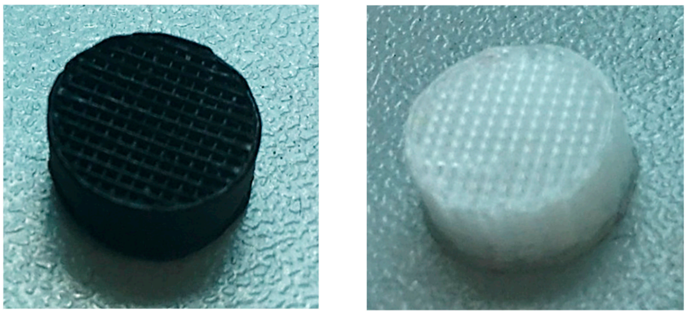

Figure S4. PLA/PD-$BaSO_4$ (left) and neat PLA (right) 3D-printed cylindric scaffolds for compression tests.

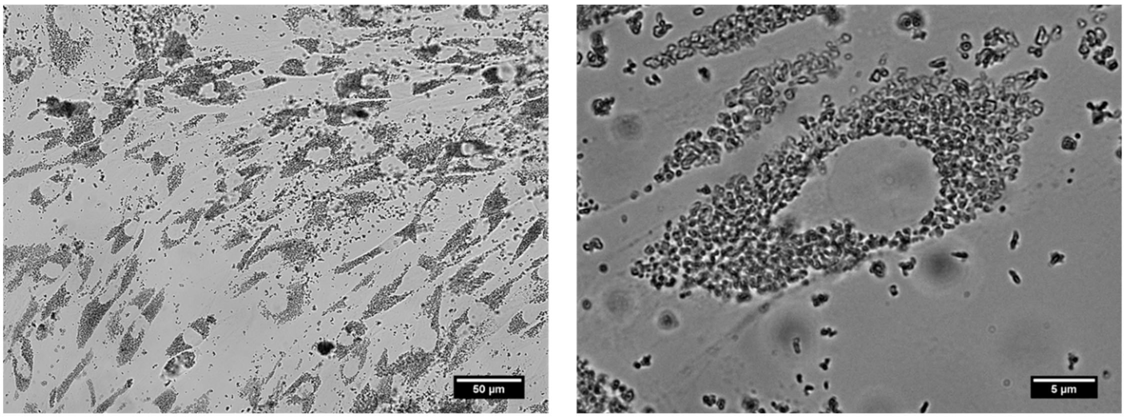

Figure S5. HDFs seeded in the presence of 500 µg/ml of $BaSO_4$.

The morphology of the cells does not change despite being in contact with the barium sulfate particles, therefore it is suggested the internalization of the particles.

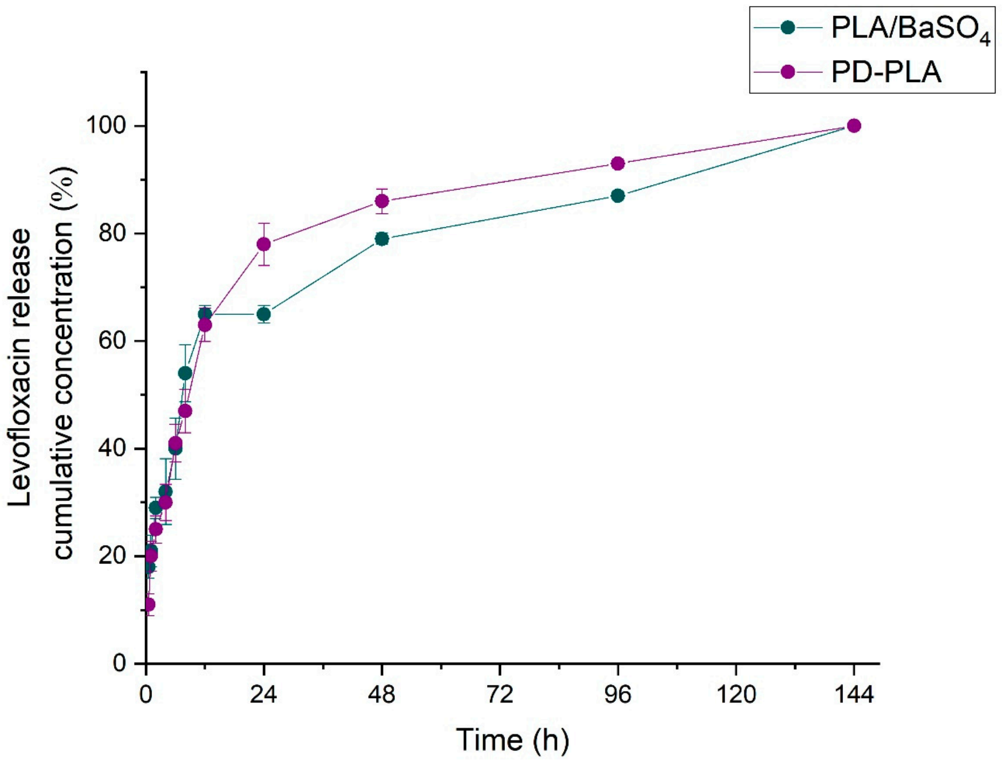

Figure S6. Release profiles over time of levofloxacin in neat PLA, PD-PLA and PLA/PD-$BaSO_4$.

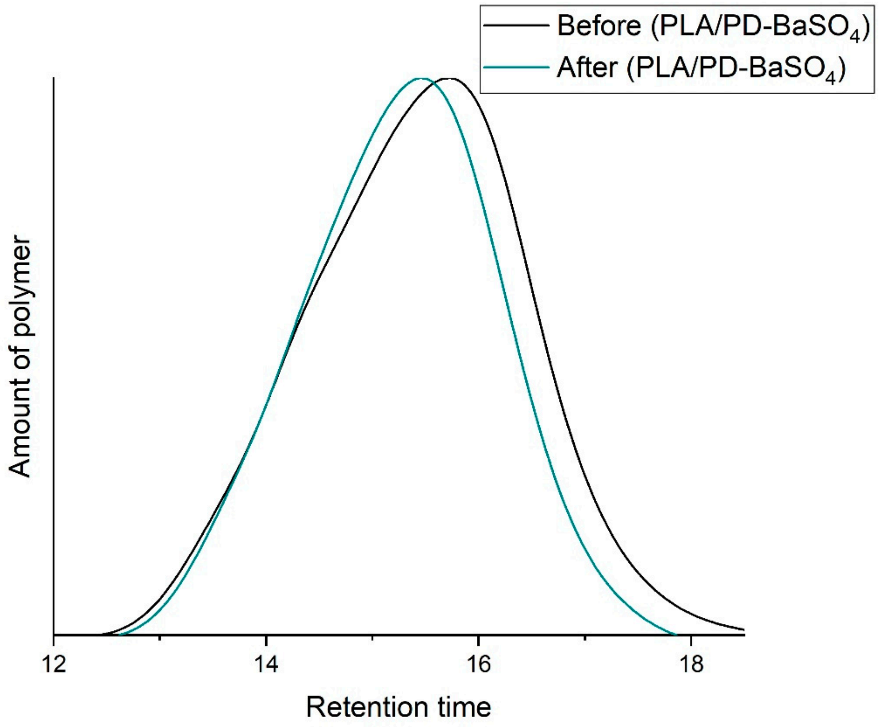

Figure S7. GPC refractive-index signals for PLA/PD-$BaSO_4$ before and after blending process at 200ºC

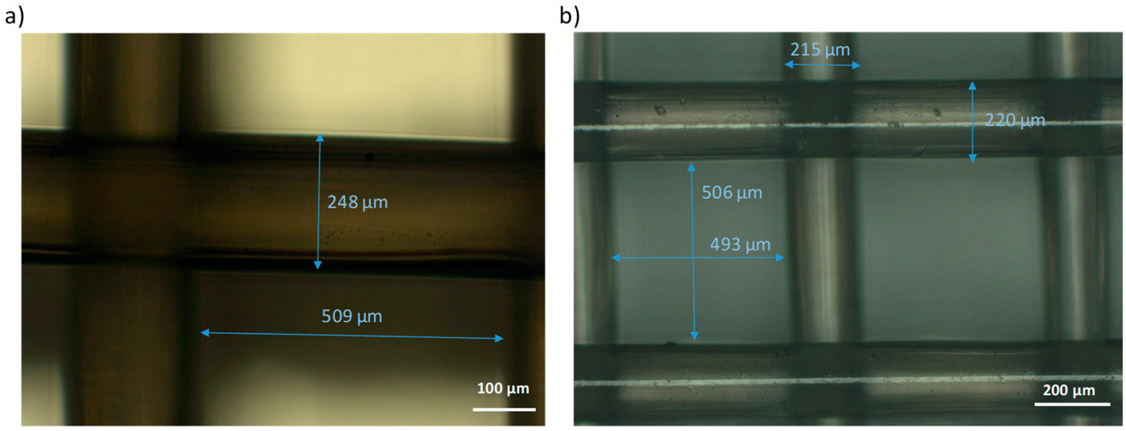

Figure S8. The microscopy image of PLA scaffold. a) x10 and b)X5

	Metabolyc activity (%)		
**Materials**	**Day 1**	**Day 3**	**Day 7**
10 µg/ml of $BaSO_4$	95 ± 3	97 ± 1	99 ± 4
50 µg/ml of $BaSO_4$	94 ± 1	93 ± 2	95 ± 3
100 µg/ml of $BaSO_4$	90 ± 2	92 ± 3	94 ± 3
500 µg/ml of $BaSO_4$	90 ± 2	91 ± 3	94 ± 2
10 µg/ml of PD-$BaSO_4$	89 ± 5	94 ± 3	95 ± 4
50 µg/ml of PD-$BaSO_4$	86 ± 4	90 ± 4	92 ± 3
100 µg/ml of PD-$BaSO_4$	89 ± 3	91 ± 3	93 ± 3
500 µg/ml of PD-$BaSO_4$	83 ± 5	90 ± 3	91 ± 3
TPC (control)	100 ± 1	100 ± 1	100 ± 2
PLA	100 ± 1	94 ± 1	100 ± 1
PLA/$BaSO_4$	98 ± 1	100 ± 1	99 ± 1
PLA/PD-$BaSO_4$	100 ± 1	96 ± 1	98 ± 1

Table S1. Summary of the metabolic activity of the $BaSO_4$ and PD-$BaSO_4$ particles as well as the materials PLA, PLA/ $BaSO_4$ and PLA/PD-$BaSO_4$.
